# Biopsychosocial Characteristics, Using a New Functional Measure of Balance, of an Elderly Population with CLBP

**DOI:** 10.3390/healthcare4030059

**Published:** 2016-08-23

**Authors:** Ryan Hulla, Michael Moomey, Tyler Garner, Christopher Ray, Robert J. Gatchel

**Affiliations:** 1 Department of Psychology, University of Texas at Arlington, Arlington, TX 76019, USA; ryanhulla@yahoo.com; 2 Department of Kinesiology, University of Texas at Arlington, Arlington, TX 76019, USA; moomey49@gmail.com (M.M.); tgarner@uta.edu (T.G.); 3 College of Health Sciences, Texas Woman’s University, Denton, TX 76204, USA; chrisray@twu.edu

**Keywords:** biopsychosocial, chronic low back pain, postural control, PROMIS, elderly patients

## Abstract

This study examined the biopsychosocial characteristics of chronic low back pain (CLBP) in an understudied but increasingly larger part of the population: the elderly (i.e., 65 years and older). A new innovative physical functioning measure (postural control, which is a proxy for the common problem of slips and falls in the elderly) was part of this biopsychosocial evaluation. Also, the National Institutes of Health (NIH)-developed Patient-Reported Outcome Measurement Information System (PROMIS) was also part of this comprehensive evaluation. Two demographically-matched groups of elderly participants were evaluated: one with CLBP (*n* = 24); and the other without (NCLBP, *n* = 24). Results revealed significant differences in most of these measures between the two groups, further confirming the importance of using a biopsychosocial approach for future studies of pain and postural control in the elderly.

## 1. Introduction

The recent Institute of Medicine Report has documented that musculoskeletal pain is the most common single type of chronic pain; chronic low back pain (CLBP) is the most prevalent in this category [1]. The economic burden of CLBP is also quite large, and continues to grow in the U.S. It should also be kept in mind that, with the “graying of America”, this CLBP problem will significantly increase in the future. Currently, there are approximately 35 million Americans 65 years or older, accounting for 12.4% of the total population [2], or about 38 million Americans [3]. By the year 2030, it is projected that about 20% of the population (72 million) will be 65 years of age or older [2]. Awareness of these population trends contributes to increased concern about healthcare issues among older adults, including CLBP. Indeed, the U.S. is in the process of what is known as the “longevity revolution”, an occurrence happening as the population of older adults increases. Making up approximately 12% of the population in the US, older adults are more susceptible to falls because of age-associated ailments [4], resulting in roughly one-third of older adults falling annually, with one-fifth of them necessitating medical attention [5]. The monetary burden associated with fall injuries (especially low back pain) is projected to reach $32.4 billion dollars by the year 2020 [6]. 

Falls and fall-related injuries (such as CLBP) are one of the chief origins of morbidity in older adults, and are a precursor to functional impairment, disability, fractures, pain and, therefore, lower quality of life [7,8]. In more severe cases, falls have been a significant cause of injury-related death in the older adult population [6]. While it is generally accepted that there is an association between falls and chronic pain [9], the relationship between falls and low-back pain from a causal perspective is not entirely understood. This study did not seek to find a causal link. However, a brief overview provides some context for the relationship between fall-risk and CLBP. Rudy, Weiner, Lieber, Slaboda, and Boston found significant differences in physical function, psychosocial function, and severity of medical comorbidity in high functioning community-dwelling older adults with CLBP compared to those who were pain free [10]. Weiner, Rudy, Morrow, Slaboda, and Lieber found a distinct relationship between neuropsychological performance, pain, and physical function [11]. Furthermore, the psychological phenomenon known as “fear of falling” may play a role in increasing the risk for subsequent falls via further limiting physical activity [12] as well as being the catalyst for changes in gait mechanics that lead to inefficient gait characteristics [13], further increasing the risk for falls. The deliberate avoidance of physical activity seen in fear of falling can also lead to muscle atrophy (a marker of physical frailty) [14], which is also seen in older adults with chronic low back pain [15]. 

Physical activity has been confirmed to improve physiological functions such as balance, flexibility, and muscle tone, thereby reducing the likelihood of sustaining a fall [16]. There is a large body of research that links physical activity, or the lack thereof, to decreased postural control and consequently increased fall risk [17]. Stubbs and colleagues report that sufficient evidence exists to conclude that exercise reduces falls in older adults. Furthermore, evidence suggests impaired physical function also impacts psychosocial well-being [17]. For example, the aging population tends to withdraw from physical activity, increasing fall risk [18]. This withdrawal from physical activity not only negatively affects an elderly individual’s postural control, but also disrupts the quality of mental well-being. In fact, Morgan and colleagues concluded that there is a strong relationship between physical activity and mental health, showing that older adults who participate in regular physical being more resistant to experiencing depression, and to having better overall mental health [19]. Overall, a large body of research shows a definite relationship between regular physical activity and improved postural control, and between regular physical activity and improved mental well-being [19], suggesting a relationship could exist between fall risk and psychosocial variables such as anxiety, depression, fatigue, pain and physical function. 

In addition, Bradbeer and colleagues found that older adults who exhibit symptoms of depression are more likely to experience chronic pain than those who are not depressed [20]. Osteoarthritis, due to physical inactivity (among other factors), contributes to chronic pain, which is then followed by avoidance of physical activity [21] thus exacerbating the cycle of decreased physical activity, sarcopenia, and osteoporosis. Chronic pain can also decrease participation in ADLs. Decreases in ADLs are seen with aging independent of pain, but pain can initiate a cycle of limited ADLs fostering a fear of movement, further decreasing functional capacity, and then to increased pain [22]. Similar to depression, anxiety is also associated with chronic pain. Older adults tend to have increased anxiety, especially when it comes to fear of falling [23], and this anxiety leads to restricted movement [24], which, as previously discussed, exacerbates pain and impairs postural control. Pain also impacts sleep quality [25], which can also directly impact postural control. One study showed that subjects who reported being “sleepy” had greater postural sway than individuals who were well-rested [26]. 

Pinpointing easily-measured variables concomitant with fall risk would be advantageous in order to reduce an individual’s risk of sustaining a fall. Because experiencing a fall can have a devastating impact on the quality of life in the elderly, it is vital that brief, inexpensive, and easy-to-use tests are available for everyday clinical use [27]. Therefore, as a first step in this process, the present study evaluated what biopsychosocial variables are related to those elderly individuals who have CLBP, and those who do not have NCLBP. Postural control, measured by the NeuroCom Balance System, and other biopsychosocial variables measured by the PROMIS-29, were evaluated. 

## 2. Methods

### 2.1. Participants

A total of 78 older adults were recruited from the local community from informative presentations about the Center for Healthy Living and Longevity (CHLL) at the University of Texas at Arlington at various places such as churches, retired faculty gatherings, word-of-mouth from friends, and even doctor recommendations. Participants took part in a research project pertaining to postural control and psychosocial assessments through CHLL at the University of Texas at Arlington. All participants had physician approval and provided informed consent to participate per the Institutional Review Board (IRB) at the University of Texas at Arlington. There were 24 participants in the CLBP group and 24 matched participants in the NCLBP group. Participants were also assessed for CLBP with the National Institutes of Health (NIH) definition of CLBP. The definition asks two questions in classifying participants with or without CLBP. They were also matched on demographic variables, which are presented in Table 1.

### 2.2. Instrumentation

The NeuroCom Smart Balance Master System detects any changes in an individual’s balance over time by measuring the participant’s ability to control the center of gravity in various sensory and motor conditions. The participant stands on a force plate, facing into a three-sided booth. The force plate and visual surround move in response to the participants’ forwards and backwards sway, creating a disturbed proprioceptive or visual input to the brain. This distortion causes the participant to rely heavily on alternative senses to maintain equilibrium. Sway refers to changes in the center of the persons applied force as a result of moving forwards or backwards. Postural control was assessed using the sensory organization test (SOT) and strategy analysis under six conditions with a NeuroCom SOT. The SOT procedure accurately identifies aberrations in the participant’s use of the three sensory systems that contribute to postural control: visual, vestibular, and somatosensory [28]. Throughout the assessment, erroneous information is delivered to the participant’s feet, eyes, and joints through “sway referencing” of the visual surround and/or the support surface. The participants were fitted with a cushioned vest that was attached to the NeuroCom’s system outer structure in order to safeguard him/her from a fall. Each condition was executed three times. Outcome measures for this test included: (1) Equilibrium Score which quantifies the center of gravity (COG) sway or postural stability; (2) Sensory Analysis ratios which are used in conjunction with the participant’s equilibrium scores to detect deficiencies of the participant’s sensory systems; (3) Strategy Analysis which measures the relative amount of movement of ankle strategy and hip strategy the participant used to maintain balance throughout each trial; and (4) COG Alignment plots the individual’s COG position at the beginning of each trial of the SOT, in which each mark determines COG alignment during a single SOT trial, relative to the center of the base of support.

After the participants’ postural control was assessed with the NeuroCom Balance System, participants were assessed for the components of physical fitness in upper- and lower-limb muscular strength and endurance, cardiovascular endurance, and upper- and lower-body muscular flexibility. Participants were then questioned on the amount of falls they have had in the past year and 6 months. No measures of physical fitness or fall frequency were used in the current study analysis. All physical fitness scorecards, NIH CLBP definition inventories, and consent forms were stored in file cabinets, and locked in the lab, and later de-identified and coded into SPSS.

The Patient Reported Outcomes Information System (PROMIS 29) is a computer-adaptive test designed to measure the following seven psychosocial constructs: physical function; anxiety; depression; fatigue; sleep disturbance; ability to participate in social roles and activities; and pain interference. The PROMIS-29 has been tested and validated for concurrent and discriminant validity, test-retest reliability, as well as participant preference for measuring health-related quality of life [29].

### 2.3. Procedures

Participants first consented to the IRB-approved protocol of the current study. After consent, participants filled out the NIH definition of CLBP inventory with paper and pencil. If the participants marked “they have had low-back pain for greater than three months or longer” and also marked “having low-back pain for at least half the days in the past 6 months or more”, the participant was classified as having CLBP. Participants who marked “having low-back pain for less than 3 months” or “having it less than half the days for the past 6 months” were classified as NCLBP.

Once consented and the NIH definition of CLBP inventory completed, and demographic information collected, the PROMIS-29 Computer Adaptive Test was administered. The computer-adaptive aspect of the PROMIS proves advantageous in that information is drawn from a large database and is formatted to a specific individual, based on the individual’s response to the previous question. The NIH is encouraging its use and have extensively developed it working towards, and achieving, validation among the population. Each participant was assigned a computer, and created a test profile before taking the assessment. When the participants finished the PROMIS-29 CAT, they logged out of their profile and results were saved, to be accessed later in order to be de-identified and transferred and coded into IBM’s Statistical Package for Social Science (SPSS). The participants also completed two other inventories on the computer after the PROMIS-29 that were not used in analysis of this present study (the Balance Efficacy Scale, and the Comprehensive Fall Risk Assessment).

### 2.4. Scoring

During the SOT, participants were tested under 6 conditions, 3 trials per condition, for a total of 18 trials. Each trial lasted 30 s. The force plate and visual surround moved in response to the participant’s center of gravity sway. The inclusive composite Equilibrium Score provides a representative score of the individual’s’ capacity to sustain postural stability throughout all conditions. Effective use of the individual’s’ sensory inputs is derived from the overall pattern of scores on each of the six conditions. The composite Equilibrium Score is the weighted average encompassing the average scores of conditions 1–6.

The Strategy Analysis score is derived by plotting the data from the force plate and the Equilibrium Scores together to quantify the amount of movement of the ankles or the hips. The Strategy Analysis score reflects the extent of movement concerning the ankles (ankle-dominant strategy) and hips (hip-dominant strategy) used to sustain postural stability throughout each trial. The closer the scores are to 100, the more ankle-dominant strategy was used to maintain stability. Conversely, the closer to 0 score reflects a more hip-dominant strategy used to maintain postural control. Typically, as stability is sustained, individuals utilize an ankle-dominant strategy primarily, shifting to a more hip-dominant strategy under conditions where postural control is more difficult to maintain [30].

The constructs of the PROMIS-29 item banks have been individually developed using patients’ representative of the 2000 US Census [29]. The subsequent question pool contrasts between each domain (anxiety, depression, fatigue, pain-interference, sleep disturbance, and physical function). There are 29 questions each in the anxiety and anger domains, 28 questions with respect to depression, 95 questions pertaining to fatigue, 41 in the pain-interference bank, 39 questions with regards to pain-behavior, 27 questions about sleep disturbances, 124 questions regarding physical function, 16 questions in the sleep impairment domain, 12 and 14 in the social impairment and social roles domains, respectively. The CAT selects a group of questions from the item pool for the participants to answer, generally 4–12 questions per domain. The CAT presents the first question and, based on the participant’s answer, selects subsequent questions from the question bank, until the responses satisfy the precision criteria of 80% reliability [29]. The resultant outcome is a t-score and standard deviation based on the standardized US population. The mean t-score is 50 and the standard deviation is 10. An individual score is given per each domain. Each domain provides a total score, a score compared with the general US population, a score compared with patients in the same age group, and a score compared with non-patients in the same age group. Each score is reported as either better or worse than norms [29].

### 2.5. Data Analyses 

SPSS version 22.0 statistical software was used to conduct all statistical analyses.

## 3. Results

The PROMIS data for each of the two groups are presented in Table 2. Multivariate statistical analysis of these data yielded a significant Pillai’s Trace Statistic of *V* = 0.40 *F*(6, 41) = 4.59, *p* = 0.001, η*_p_*^2^ = 0.40. As can be seen, CLBP and NCLBP groups significantly differed (based on separate analyses of variance) for: perception of pain interference, *F*(1, 46) = 24.89, *p* < 0.001, η*_p_*^2^ = 0.35; perception of physical function, *F*(1, 46) = 10.26, *p* = 0.002, η*_p_*^2^ = 0.18; and fatigue *F*(1, 46) = 5.01, *p* = 0.03, η*_p_*^2^ = 0.10. Sleep disturbance approached significance *F*(1, 46) = 3.01, *p* = 0.089, η*_p_*^2^ = 0.06. It should be noted that all of the above had medium-large effect sizes (large: >0.14; medium: >0.05; small: >0.009). No significant differences were found between groups for anxiety and depression.

The NeuroCom data for each of the two groups are presented in Table 3. Multivariate statistical analysis of the data did not yield statistical significant results between CLBP and NCLBP groups’ overall equilibrium, strategy (ankle-dominant or hip-dominant strategy), or composite balance scores. However, for differentiating between CLBP versus NCLBP groups, the following measures taken together were significant: NeuroCom average equilibrium scores in conditions four, five, and six; NeurCom average strategy scores in condition three and six; overall average NeuroCom somatic system score; and PROMIS scores on pain inference and sleep disturbance, *R*^2^ = 0.56, *F*(8, 39) = 6.15, *p* < 0.001. Results of this regression model yielded individual significant relationships, reported as individual beta-weight t tests, for: average scores of equilibrium NeuroCom condition four (β = −0.29, *t*(39) = −2.02, *p* = 0.05); equilibrium average score in NeuroCom condition five, (β = 0.50, *t*(39) = 2.39, *p* = 0.022); NeuroCom overall somatic system score (β = −0.25, *t*(39) = −2.26, *p* = 0.029); sleep disturbance (β = 0.31, *t*(39) = 2.45, *p* = 0.019); and pain interference (β = 0.45, *t*(39) = 3.56, *p* = 0.001). Average strategy score on NeuroCom condition three approached statistical significance (β = −0.28, *t*(39) = −1.98, *p* = 0.054).

## 4. Discussion

The purpose of this present study was to determine whether or not a relationship exists between a new functional measure of balance (postural control as assessed using the NeuroCom Balance System), and the PROMIS psychosocial variables, in elderly individuals with or without CLBP. A number of significant findings were revealed. Most importantly, there were differences found between the two groups on various psychosocial measures and newer postural control functioning indices. To date, there has been little to no research conducted to establish whether or not a relationship exists between postural control and mental health and well-being, especially in the elderly. Moreover, the logistic regression model independently replicated a number of previous studies that assessed only one or two of the measures evaluated in the present more comprehensive biopsychosocial investigation. For example, Bradbeer and colleagues have found that older adults who experience symptoms of depression are more likely to exhibit chronic pain than older adults who are not depressed [20]. A number of other studies have independently confirmed some of the individual associations revealed in the present investigation. For example, Messier and colleagues found osteoarthritis contributed to chronic pain, avoidance of physical activity, sarcopenia, and osteoporosis [21]. Mossey and Gallagher reported that pain initiated a decreased ADL, fostering a fear of movement, and decreasing functional capacity [22]. Howland and colleagues showed that older adults tend to have increased anxiety, particularly in regards to fear of falling [23], van Haastregt and colleagues reported that anxiety leads to limited movement [24]. Lautenbacher, Kundermann, and Krieg found that pain impaired sleep quality [25]. Finally, Jorgensen and colleagues revealed that being “more sleepy” resulted in greater postural sway [26]. The great significance of the present investigation is that it is the only one in the scientific literature to evaluate the majority of the measures reported in the aforementioned studies together as a whole in an elderly population, and differentiating those participants who either had CLBP or NCLBP.

The field of biopsychosocial clinical research views the importance of the interaction among biological, psychological and social factors in pain, and the need in taking all of these into consideration when evaluating the “whole” person [31,32]. The significance of the present study was the use of a relatively new physical measure of postural control, and its relationship to pain and other psychosocial measures (as assessed by the PROMIS) in an elderly community-dwelling population. Taken as a whole in the regression model, it was revealed that there were greater levels of perceived pain inference, sleep disturbance, and fatigue (in the CLBP sufferers) compared to their NCLP counterparts. Also, there were significantly lower scores on perceived physical function and strategy of balance in the CLBP group, relative to the NCLBP group. Moreover, the CLBP group had greater scores on depression and anxiety, with lower scores in equilibrium and composite balance compared to the NCLBP group.

Of course, it should be noted that in any clinical research study of this type, there could be some potentially confounding factors that may or may not have played a role in influencing the findings. For example, the selection process of the participants in the sample could be a source of bias [32] due to the sample not being representative of the population in terms of education level and income level, history of diseases among participants, and medication influence. In the total matched paired sample of the current study, 73.1% of the participants had a college degree, and 38.5% of the total sample had a graduate or professional degree. It has been reported that individuals with lower levels of education are more likely to have a sedentary lifestyle, relative to those with a higher education. There are many health-risk factors and unhealthy habits associated with a sedentary lifestyle [22]. The sample of participants in the current study were more educated than the normal population and, therefore, may not have been totally representative of the population as a whole. Nevertheless, as reviewed above, many novel and important statistically significant findings were revealed, and the study provided the first comprehensive biopsychosocial results in the scientific literature, using different outcome measures, in the under-studied elderly population with CLBP. These results warrant further investigation.

## 5. Conclusions

The results of the study yielded significant differences between elderly individuals with CLBP and NCLBP, with the CLBP participants scoring higher in the psychosocial dimensions of pain interference, fatigue, and the approaching significance in regards to the dimension of sleep disturbance. Physical function scores were also significantly different between groups, with the CLBP group scoring lower than the NCLBP group. No significant differences were found between groups in regards to balance variables measured by the NeuroCom balance system, although the variables of condition four results of the NeuroCom (participant balance on a tilting force plate from sway with eyes closed), equilibrium average scores on condition five (force plate and surroundings move in regard to participants sway), NeuroCom overall somatic scores, along with sleep disturbance and pain interference measures, significantly predicted CLBP among participants, with strategy scores on condition three of the NeuroCom approaching significance as a predictor of CLBP. The results suggest that it is imperative that a biopsychosocial approach is used when investigating future constructs for the manifestation and management of pain and fall prevention in the geriatric population, and suggest a treatment to address the psycho-social and balance aspects of CLBP.

## Figures and Tables

**Table 1:** Demographics.

Measure	CLBP	NCLBP	Matched Pair (Total)
Sample size	24	24	48
Mean Age	73.96	74.04	74.00
Male	33.3%	33.3%	33.3%
Female	66.7%	66.7%	66.7%
Previously Exercised (Yes)	58.3%	50.0%	54.2%
Previously Exercised (No)	41.7%	50.0%	45.8%
Education Less than 9th Grade	0.0%	0.0%	0.0%
High School Graduate/GED	4.3%	4.3%	4.3%
Some College	34.86%	26.10%	30.4%
Associates Degree	4.3%	8.7%	6.5%
Bachelors Degree	39.1%	30.4%	34.8%
Graduate/Professional Degree	17.4%	30.4%	23.9%

**Table 2:** Patient-Reported Outcome Measurement Information System (PROMIS) data descriptives.

Measure	CLBP		NCLBP	
	M	SD	M	SD
Anxiety	51.38	6.63	50.25	7.30
Depression	49.08	5.33	46.54	6.29
Fatigue	53.00	7.60	48.71	5.53
Pain Interference	59.17	7.15	49.08	6.85
Physical Function	40.96	4.91	46.29	6.51
Sleep Disturbance	48.38	5.82	45.33	631

**Table 3:** Neurocom data descriptives.

Measure	CLBP		NCLBP	
	M	SD	M	SD
Strategy	81.85	6.55	84.88	4.96
Equilibrium	77.32	5.34	79.14	5.41
Composite Balance	73.96	6.53	76.00	6.99
